# The balance of metagenomic elements shapes the skin microbiome in acne and health

**DOI:** 10.1038/srep39491

**Published:** 2016-12-21

**Authors:** Emma Barnard, Baochen Shi, Dezhi Kang, Noah Craft, Huiying Li

**Affiliations:** 1 Department of Molecular and Medical Pharmacology, Crump Institute for Molecular Imaging, David Geffen School of Medicine, UCLA, Los Angeles, California, USA; 2 Los Angeles Biomedical Research Institute at Harbor-UCLA Medical Center, Torrance, California, USA; 3 UCLA-DOE Institute for Genomics and Proteomics, Los Angeles, California, USA

## Abstract

Studies have emphasized the importance of disease-associated microorganisms in perturbed communities, however, the protective roles of commensals are largely under recognized and poorly understood. Using acne as a model disease, we investigated the determinants of the overall virulence property of the skin microbiota when disease- and health-associated organisms coexist in the community. By ultra-deep metagenomic shotgun sequencing, we revealed higher relative abundances of propionibacteria and *Propionibacterium acnes* phage in healthy skin. In acne patients, the microbiome composition at the species level and at *P. acnes* strain level was more diverse than in healthy individuals, with enriched virulence-associated factors and reduced abundance of metabolic synthesis genes. Based on the abundance profiles of the metagenomic elements, we constructed a quantitative prediction model, which classified the clinical states of the host skin with high accuracy in both our study cohort (85%) and an independent sample set (86%). Our results suggest that the balance between metagenomic elements, not the mere presence of disease-associated strains, shapes the overall virulence property of the skin microbiota. This study provides new insights into the microbial mechanism of acne pathogenesis and suggests probiotic and phage therapies as potential acne treatments to modulate the skin microbiota and to maintain skin health.

The skin is the largest organ in the human body and functions as the first line of defense by providing a protective barrier between the environment and inner body. The skin harbors several hundreds of resident microorganisms, which function in communities and protect the body from invasion of pathogens^1^,^2^. Several studies have shown that shifts in the skin microbiota are associated with various skin diseases^3^,^4^,^5^,^6^,^7^.

Acne vulgaris (commonly called acne) is the most common skin disease, affecting 80–85% of the population^8^,^9^. It is most prevalent in adolescents and rarely occurs in people over the age of 50^10^,^11^. Although acne is not life threatening, it can lead to severe pain and scarring on the skin, and has profoundly negative psychosocial effects^12^,^13^. Acne is a disease of the pilosebaceous unit (commonly known as the hair follicle)^14^. While its etiology is still unclear with multiple factors involved, the Gram-positive lipophilic anaerobe *Propionibacterium acnes* has been thought to play a role in acne pathogenesis^15^,^16^. On the other hand, *P. acnes* is believed to contribute to skin health, preventing the colonization of opportunistic pathogens via its ability to convert sebum to free fatty acids and to maintain an acidic skin pH^17^,^18^.

In our previous characterization of the skin microbiome in healthy individuals and acne patients using 16S ribosomal RNA (rRNA) analysis, we revealed that certain *P. acnes* strains were highly associated with acne while some strains were associated with healthy skin^5^. At the genome level, *P. acnes* strains from different lineages harbor various genetic elements associated with either disease or health^5^,^19^,^20^. As individuals often harbor multiple *P. acnes* strains simultaneously which may have different roles in disease or health, it is unclear how the colonization of multiple strains in the follicle may affect the overall virulence or health properties of the skin microbial community.

Microbiome analyses based on the 16S rRNA or other phylogenetic marker genes and elements are often limited in providing molecular insight of disease association. Metagenomic shotgun sequencing analysis reveals not only the taxonomic composition but also the functional potential of the microbiome, and thus can provide better understanding of the potential microbial involvement in health or disease at the molecular level. To date, in addition to the Human Microbiome Project (HMP)^21^, only a few metagenomic shotgun sequencing analyses of the skin microbiome have been described in young healthy adults^22^,^23^. While these studies focused on the healthy skin microbiome, few studies have characterized the microbiome in skin diseases using metagenomic shotgun sequencing. The metagenomic landscape of the skin microbiome in the human population, therefore, needs to be further investigated in both healthy and disease states with an expanded age range.

To investigate the role of the skin microbiota in acne, in this study we performed a metagenomic shotgun sequencing analysis of the skin microbiota of healthy individuals and acne patients. As older individuals rarely develop acne, we also characterized the skin microbiome of healthy individuals over the age of 55 as a comparison. The microbiome signatures for diseased and healthy states were identified and used to determine the clinical status of samples collected from healthy individuals and acne patients.

## Results

### High abundance of propionibacteria in the follicular microbiota

**Figure 1:**
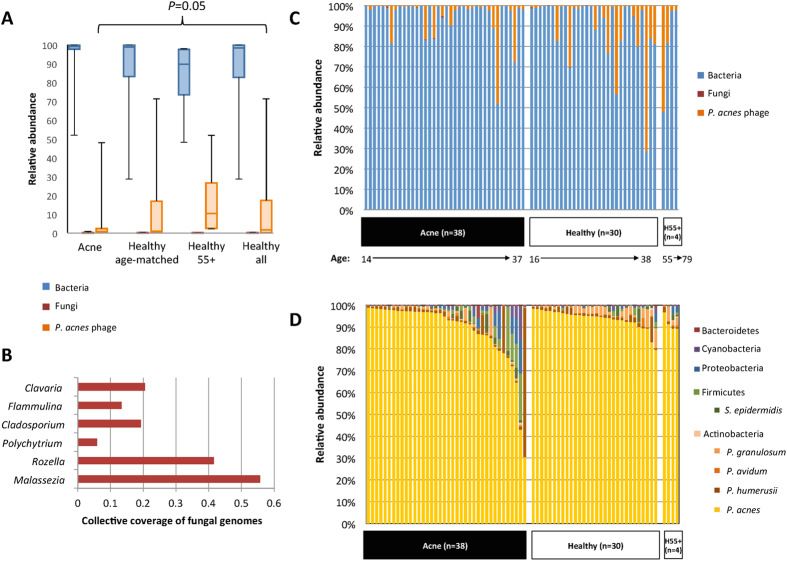
Bacteria dominate the skin follicular microbiome. (**A**) A box-and-whiskers plot comparing the relative abundances of bacteria, fungi, and *P. acnes* phage in the follicular microbiota of acne patients (n = 38), age-matched healthy individuals (n = 30), healthy individuals over 55 (H55 + ; n = 4), and all healthy individuals combined (n = 34). (**B**) A few fungal organisms were found in the follicle. Sequencing reads pooled from all subjects mapped to six fungal species, with less than 1X coverage for any species. (**C**) The relative abundance of *P. acnes* phage in all the samples suggests an increased prevalence and abundance of *P. acnes* phage in healthy individuals and a trend of increased phage abundance with age. (**D**) The relative abundances of bacterial species in the follicle. Each column represents the relative abundances of the bacterial species found in each individual. *P. acnes* was the dominant skin bacterium in all but one individual. On average *P. acnes* accounted for 91% of the bacterial taxa identified. An increase in the average relative abundances of *P. acnes* and *P. granulosum* was observed in the healthy individuals, whereas an increase in the average relative abundances of minor taxa was observed in the acne group. Five major skin bacterial species (*P. acnes, P. humerusii, P. avidum, P. granulosum, and S. epidermidis*) are shown separately from the phyla that they belong to.

To identify the disease- and health-associated microbial elements in the follicular microbiota, we performed ultra-deep metagenomic shotgun sequencing of the samples collected from 38 acne patients and 30 age-matched healthy individuals (Fig. S1). After removal of human DNA sequences and low-quality reads, we obtained an average of 1.08 gigabase pairs (Gbp) per sample (6.9 × 10^7^ bp – 4.8 × 10^9^ bp), sufficient to cover the microbial diversity of skin samples^24^. We mapped the cleaned sequencing reads to our reference genome set, which consists of 1,252 bacterial and 272 fungal genomes from the HMP reference genome database and several additional genomes of skin microorganisms: *Propionibacterium avidum*^25^,^26^*, Propionibacterium granulosum*^26^*, Propionibacterium humerusii*^27^, and *P. acnes* bacteriophage^28^,^29^. To cover different *P. acnes* strains, we also included the *P. acnes* pan-genome in the reference^19^. Consistent with other skin sites^23^, bacteria were the major organisms found in the follicle (Fig. 1A) with few fungal organisms detected at low relative abundances (Fig. 1B,C). Five main bacterial phyla were found in the samples, including Actinobacteria (95.6%), Firmicutes (2.3%), Proteobacteria (1.2%), Cyanobacteria (0.6%), and Bacteroidetes (0.2%). The dominance of Actinobacteria in the follicle is consistent with previous taxonomic analyses of sebaceous skin sites^5^,^23^,^30^.

At the species level, *P. acnes* was the most prevalent and abundant. It was found in all 68 individuals with an average relative abundance of 91.0% (Fig. 1D; Table 1). The healthy individuals had a slightly higher relative abundance of *P. acnes* than the acne patients (93.8% *vs.* 88.5%). We also compared the differences in *P. acnes* strain populations between acne patients and healthy individuals. The relative abundances of the dominant *P. acnes* strains were assigned based on the read coverages of the 16S rRNA SNPs that distinguish the ten most common (top ten) *P. acnes* ribotypes (RTs)^5^ (Fig. S2). We found that acne patients had a higher diversity of *P. acnes* population (Shannon index = 1.07) with more RTs (2.55 RTs per individual) than healthy individuals (Shannon index = 0.79, *P* = 0.039; 1.97 RTs per individual, *P* = 0.007). Consistent with our previous study^5^, RT4, RT5, and RT8 were found to be more abundant and more prevalent in acne patients, with RT4 being statistically significantly different in relative abundance (*P* = 0.004), while RT2 and RT6 were more abundant in healthy individuals (Fig. S2). The strain association with acne or healthy skin is consistent with previous multi-locus sequence typing and 16S ribotype analysis of large collections of clinical *P. acnes* isolates^5^,^31^,^32^.

Other cutaneous *Propionibacterium* species, including *P. humerusii* (2.7%), *P. granulosum* (1.6%), and *P. avidum* (0.4%), were also detected (Table 1). Among them, *P. granulosum,* a resident bacterium of sebaceous skin sites^33^, was significantly more abundant in the healthy individuals than in the acne patients (*P* = 0.002). 

Other bacterial species frequently identified in the follicle microbiota include *Staphylococcus epidermidis* (0.9%), *Staphylococcus capitis* (0.4%), *Escherichia coli* (0.7%), and *Clostridium sp.* (0.5%). Minor bacterial taxa were found more prevalent and abundant in acne patients than in healthy individuals with varying presence and abundance (Supplementary Text). On average, acne patients had a higher diversity at the species level than healthy individuals, but not statistically significantly different (Shannon index = 0.63 and 0.44, respectively; *P* = 0.072). Reduced *P. acnes* and *P. granulosum* and increased minor taxa observed in acne patients suggest disruptions of the commensal skin flora in acne development.

### The skin microbiome of older healthy individuals is similar to younger healthy individuals

Acne rarely occurs in individuals over the age of 50^10^,^11^. The skin microbiome profiles of older individuals with clear skin can be used as independent references for healthy, acne-free skin. However, the skin metagenome of older healthy individuals has not been characterized. We performed metagenomic shotgun sequencing of the samples collected from four healthy individuals of age 55–79 (Fig. S1). The bacterial compositions of the follicular microbiome of these individuals were similar to those of the younger healthy individuals (Fig. 1D). It was previously noted that the production of sebum, a nutrient source for lipophilic bacteria, including *Propionibacterium* species, decreases with age^34^. In our analysis we found that the relative abundances of both *P. acnes* and *P. granulosum* were higher in older healthy individuals than in acne patients (Table 1), similar to the observations seen in younger healthy individuals. When grouping all healthy individuals together, *P. granulosum* remained significantly more abundant in healthy skin than acne (*P* = 0.001). At the strain level, *P. acnes* populations in older healthy individuals were similar to those of the younger healthy cohort, consisting of mostly RT1, RT2, and RT3 strains, with little or no RT4, RT5, and RT8 strains detected (Fig. S2).

When comparing all 34 healthy individuals, including the four subjects over the age of 55, with the 38 acne patients, we found an increased prevalence and abundance of *P. acnes* phage in the healthy group (*P* = 0.05) (Fig. 1A). We also observed a trend between age and the relative abundance of *P. acnes* phage. Higher relative abundances of phage were found more often in individuals with increased age (Fig. 1C).

### Metagenomic composition of the skin microbiome is different between acne patients and healthy individuals

To better understand the role of the follicular microbiota in acne pathogenesis at the molecular level, we investigated the differences in metagenomic composition of the microbiome between acne patients and healthy individuals. Among the sequencing reads mapped to *P. acnes,* we identified 2,707 operational gene units (OGUs) in the samples, among which 1,943 OGUs (72%) were present in every sample. Rarefaction analysis indicated that the sequencing depth was sufficient to quantitatively compare the *P. acnes* functional profiles between acne patients and healthy individuals (Fig. S3). Since the microbiome profiles were similar between younger and older healthy individuals at both the species and *P. acnes* strain level, we combined the healthy individuals from both age groups in the following metagenomic analyses.

To determine whether specific metagenomic elements are associated with acne or healthy skin, we compared the relative abundances of *P. acnes* OGUs between acne patients and healthy individuals (Fig. S4). As the skin microbiome varies greatly among individuals^23^,^30^,^35^,^36^, to reduce the potential bias due to specific individuals recruited in our study, we randomly selected 100 subsets of the data, each containing two-thirds of the individuals from each clinical group (26 acne patients and 22 healthy individuals), and determined the metagenomic elements that were differentially abundant between the two groups in each random set. To capture the differences at the species level between the two cohorts, in addition to *P. acnes* OGUs, we also considered the species found in the samples as additional metagenomic elements and included them in the analysis. To determine the most robust set of metagenomic elements that are associated with either acne or healthy skin, we identified 63 metagenomic elements, whose relative abundances were significantly different (*P* < 0.05) between the two groups in at least 50 of the 100 random subsets. These 63 elements consisted of 62 *P. acnes* OGUs (Table S1) and *P. granulosum* as species.

Among the 62 *P. acnes* OGUs, 25 were significantly more abundant in acne patients. Two of them are involved in thiopeptide bacteriocin precursor synthesis and transport (PAGK2104 and PAGK2105, as annotated in *P. acnes* HL096PA1 genome). Thiopeptide bacteriocins belong to a family of microcins, which are produced by Gram-positive bacteria and inhibit the growth of other Gram-positive species by blocking protein translation^37^. Additionally, 19 of the 25 acne-associated OGUs were from locus 2 (PAGK0160-PAGK0178), a genomic island previously identified mainly in RT4 and RT5 strains and highly associated with acne^5^,^20^. Locus 2 spans over 20 Kb and encodes 23 ORFs (Fig. S5). The differentially abundant locus 2 OGUs identified include a number of genetic elements involved in recombination, such as a single-strand binding like protein (Ssb), shown in other bacterial species to be involved in chromosomal transformation^38^, and resolvase-like protein, previously implicated in genetic integration. Other OGUs include a cluster of Streptolysin S-associated genes (*sag*) involved in the biosynthesis and transport of a bacterial toxin. Identified and characterized in *Streptococcus pyogenes, sag* genes are involved in synthesis, post-translational modification, and transport of a ribosomally synthesized bacteriocin, which has been linked to antimicrobial activity as well as invasive infections^39^,^40^. The presence of genes homologous to *sagB, C*, and *D* in locus 2 suggests involvement of this locus in bacteriocin biosynthesis and maturation. Additionally self-immunity and transport genes are found immediately downstream. Other OGUs in this locus have putative roles in cell viability, virulence, and immunity including ATP-binding cassette (ABC) transporter and ABC-binding protein for translocation of lipids, nutrients and/or toxins, CAAX amino protease, which is thought to be involved in self-immunity^41^, and partitioning machinery needed for cell replication and division. While the specific functions of genes in this locus in acne are unclear, the absence of locus 2 in healthy individuals and a high abundance in acne patients provides strong evidence for its association with acne.

**Figure 2:**
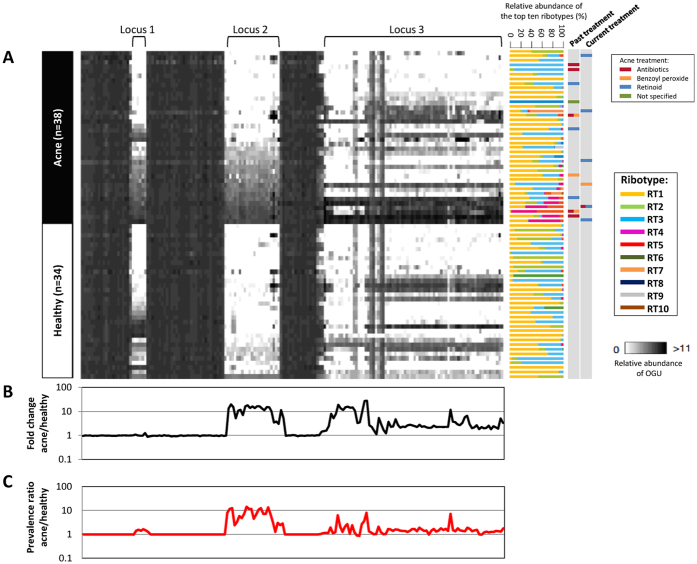
Differences in the relative abundances of *P. acnes* OGUs between acne patients and healthy individuals. (**A**) A heat map showing the relative abundances of the OGUs in *P. acnes* loci 1, 2, and 3 in acne patients and healthy individuals. Each column represents an OGU, ordered based on the genomic location of the OGUs. OGUs 101–200 in the pan-genome were plotted to show locus 1, flanking OGUs, and locus 2. The 74 OGUs from locus 3, which is a plasmid, are also shown. Each row represents an individual. Acne patients (n = 38) and healthy individuals (n = 34, including those with age over 55) were compared. Individuals within each group were clustered based on the average relative abundance of locus 2 OGUs. Ribotype composition and past and current acne treatments are indicated on the right. Multiple treatments are depicted by more than one color. (**B**) Fold changes in relative abundance of the OGUs in loci 1, 2, and 3 between acne patients and healthy individuals. Acne-associated OGUs had a fold change >1, while health-associated OGUs had a fold change <1. (**C**) Prevalence ratio of the OGUs in loci 1, 2, and 3 between acne patients and healthy individuals. The presence of an OGU in a sample is defined as an OGU with at least 1X coverage after normalization.

In addition to locus 2, in our previous studies, two other genomic loci (locus 1 and locus 3) were found enriched in acne-associated RT4 and RT5 strains^5^,^19^,^20^. To examine whether these three loci were overrepresented in the metagenome of acne patients, we plotted the relative abundance (Fig. 2A), the fold change between acne patients and healthy individuals (Fig. 2B), and the prevalence ratio (Fig. 2C), of each OGU encoded in these three loci across all individuals. We found a higher average relative abundance and prevalence of all three loci in acne patients compared to healthy individuals (Fig. 2). Locus 2 was rarely found in the microbiome of the healthy individuals, and was significantly associated with acne (*P* = 0.002) (Fig. 2). Since most RT4 and RT5 strains harbor locus 2 and are resistant to antibiotics^5^,^19^, we examined the treatment history of each individual to determine whether the presence of locus 2 was due to acne treatment. While locus 2 was found in some treated acne patients (n = 8), it was absent in other treated patients (n = 8) and was present in many untreated patients (n = 10). The presence of locus 2 was not significantly different between treated and untreated patients (Fisher’s exact test, *P* = 1), suggesting that locus 2 is a characteristic of the disease rather than treatment (Fig. 2A). Additional analysis to further support the association of locus 2 with the disease is described in the supporting text (Fig. S6; Supplementary Text).

Of the 62 differentially abundant *P. acnes* OGUs, 37 OGUs were more abundant in healthy individuals. In contrast to the enrichment of virulence-related genes observed in the acne metagenome, genes involved in microbial metabolism and nutrient biosynthesis were significantly more abundant in the healthy metagenome (Fig. S4). Examples include glycosyl transferase (PAGK0136), D-alanine–D-alanine ligase (PAGK0821), and cobalamin-independent methionine synthase (PAGK1035), which are involved in polysaccharide, cell wall, and amino acid biosynthesis, respectively. An enrichment of these genes in the healthy metagenome is consistent with the metagenomic study by Mathieu *et al*.^22^, which suggested a functional role of the resident bacteria in healthy skin in the exploitation of compounds from the human skin including sugars, lipids, and iron^22^. Other significantly more abundant OGUs in the healthy metagenome include glycerol uptake facilitator protein (PAGK2214), which aids in carbohydrate metabolism processes by transporting glycerol across the cytoplasmic membrane. Recently it was shown that fermentation of glycerol by *P. acnes* contributes to skin and host health via the production of short chain fatty acids (SCFA)^42^. SCFA including acetic acid, lactic acid, and propionic acid were shown to significantly decrease the colonization of skin-associated pathogens such as methicillin-resistant *Staphylococcus aureus*^42^.

### The balance between acne- and health-associated metagenomic elements shapes the host skin microbiota in acne and health

**Figure 3:**
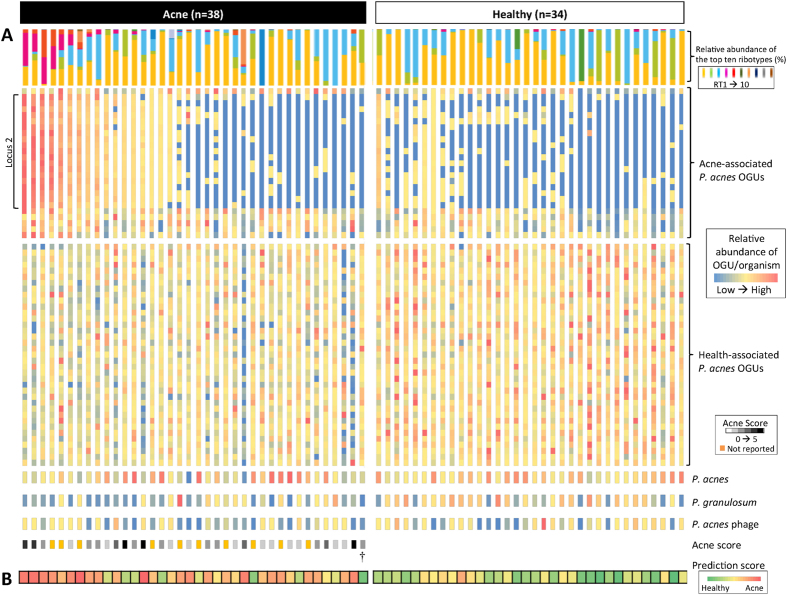
The relative abundances of acne- and health-associated metagenomic elements in acne and healthy individuals. **(A**) The relative abundances of 62 *P. acnes* OGUs, including 25 acne- and 37 health-associated OGUs, and three organisms associated with healthy skin, *P. acnes, P. granulosum*, and *P. acnes* phage, were plotted for each individual to illustrate the importance of a balance between these metagenomic elements in health and acne. Each column represents an individual, and each row represents an OGU or an organism. The top ten ribotype composition and acne severity score (acne patients only) of each individual are also shown. (**B**) The prediction score of each individual based on the relative abundances of the 45 metagenomic elements is shown, where red indicates acne and green indicates healthy skin. The classification of the clinical states had an overall accuracy of 85%.

Our observations of differentially abundant species, strains, and metagenomic elements between the skin microbiome of acne patients and healthy individuals led us to hypothesize that the balance between acne- and health-associated metagenomic elements determines the virulence and health properties of the microbiota in skin disease and health. We define the balance as the ratio between the relative abundances of acne- and health-associated metagenomic elements. When the relative abundances of the 62 *P. acnes* OGUs and *P. granulosum* from all individuals were compared, we found that this ratio in acne patients was significantly higher than in healthy individuals (*P* = 2.9 × 10^−5^). Figure 3 illustrates the relative abundances of these 63 metagenomic elements across all 72 individuals. The metagenomic profiles of healthy individuals were readily distinguishable from acne patients harboring locus 2. Acne patients with locus 2 had significantly higher relative abundances of acne-associated OGUs (in 24 of 25 OGUs *P* = 0.00024–0.021, and in 1 of 25 OGUs *P* = 0.083) (Fig. 3A). However, these subjects only represented about half (47%) of the patients recruited in our study. When we examined the metagenomic profiles of the other acne patients without locus 2, we found that they were distinct from healthy individuals with decreased relative abundances of health-associated *P. acnes* OGUs (in 23 of 37 OGUs *P* = 0.0024–0.046, and in 14 of 37 OGUs *P* = 0.058–0.30). We were able to reveal distinct microbiome profiles between acne patients and healthy individuals that were not readily apparent at the taxonomic level, with differences in the relative abundances of the metagenomic elements that were associated with either acne or healthy skin (Fig. 3).

In addition to *P. acnes* OGUs, we found that the relative abundances of *P. acnes* and *P. granulosum* were also important factors influencing the clinical state of the skin. As an example, an acne patient in our cohort (labeled with † on Fig. 3) displayed a profile of the 62 *P. acnes* OGUs that was more similar to the healthy individuals than all other acne patients (*P* = 5.29 × 10^−5^). However, the observed low relative abundances of both *P. acnes* (78% compared to the average of 91%) and *P. granulosum* (0.070% compared to the average of 1.6%) may contribute to the acne status of this patient. This suggests that a healthy follicular microbiota not only requires health-associated *P. acnes* strains and genetic elements, but also requires significant dominance of resident propionibacteria. Taken together, our findings suggest that the balance between skin metagenomic elements determines the virulence and health properties of the skin microbiota and is important in skin health and disease.

**Figure 4:**
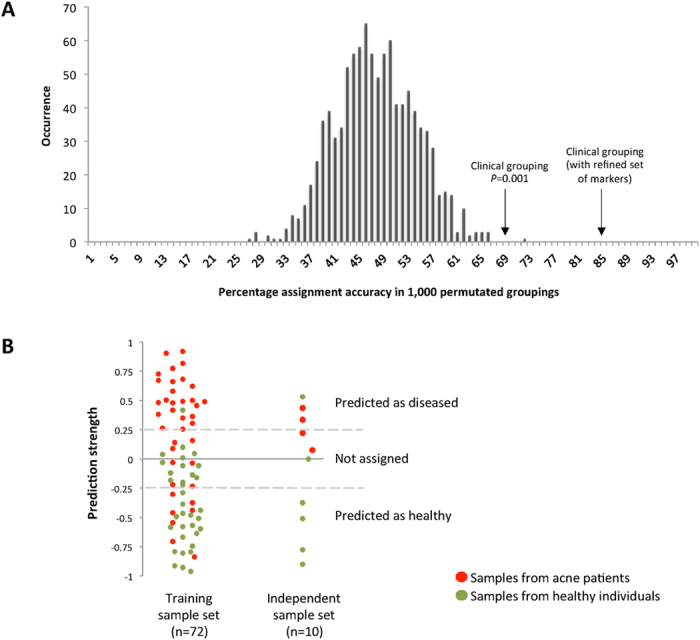
Class prediction accuracy using leave-one-out cross-validation and weighted gene-voting. (**A**) For the training sample set (n = 72), using the clinically defined acne and healthy individual grouping, the classifier correctly assigned the clinical states of 34 of the 49 assigned samples (69% accuracy) using a prediction strength threshold of 0.25. This result is statistically significant (*P* = 0.001), because only one of the 1,000 permutated groupings had a higher accuracy. However, that particular grouping (accuracy of 72%) had fewer samples assigned than the clinical grouping (n = 39 vs 49). This demonstrates that the differences in the relative abundances of the metagenomic elements between acne patients and healthy individuals can be used to predict the clinical states of the skin. When we used the refined set of 45 metagenomic elements, including 43 *P. acnes* OGUs, *P. acnes* locus 2, and *P. granulosum*, we further improved the prediction accuracy of the training sample set to 85%. (**B**) Consistent with the prediction accuracy on the training sample set, for the independent sample set (n = 10), the 45 metagenomic elements were able to assign 70% of the samples with 86% accuracy.

We further investigated whether the relative abundance profiles of the metagenomic elements are sufficiently robust to correctly classify the clinical state of skin samples. We performed a supervised class prediction analysis based on a modified weighted gene voting algorithm^43^,^44^ (Supplementary Text). We generated 1,000 permutations. In each permutation, samples were randomly assigned to either acne or healthy state, with 38 samples in the acne group and 34 samples in the healthy group. We identified differentially abundant metagenomic elements between the two groups among all but one sample, and predicted the clinical state of the withheld sample based on the abundance profiles of the metagenomic element, known as the leave-one-out cross-validation (LOOCV). With the use of the clinically defined acne-healthy grouping, the algorithm predicted the clinical states of 49 out of 72 samples (68%) with a prediction strength threshold of 0.25. Thirty-four of the 49 samples were assigned correctly (69% accuracy). The number of correctly assigned samples based on the clinical grouping was higher than most of the permutated groupings (*P* = 0.064). The clinical grouping also had a significantly higher prediction accuracy (69%) than all but one of the permutated groupings (*P* = 0.001) (Fig. 4A).

We further improved our classifier by using the 63 metagenomic elements, which are the most robust set based on the 100 random samplings as described earlier. Since the 19 OGUs from locus 2 had similar abundance profiles across all samples, to avoid overweighing locus 2 in the classification, we combined these 19 OGUs and used their average relative abundance in the classifier. The refined classifier therefore consisted of the abundance profiles of 45 metagenomic elements: *P. acnes* OGUs/loci, and *P. granulosum.* Based on this refined set, we were able to assign the clinical states of 31 of the 38 acne patients (82%, prediction strength threshold of 0.25). Twenty-four acne patients were correctly assigned (accuracy of 77%). Among the healthy individuals, we were able to assign 21 of the 34 subjects (62%) with 20 correctly assigned (accuracy of 95%). Overall, the classifier was able to assign 72% of the subjects with an accuracy of 85% (Fig. 3B; Fig. 4B).

Furthermore, to validate that these metagenomic elements can be used as markers for clinical classification, we collected an “independent sample set” from ten additional subjects, including 4 acne patients and 6 healthy individuals, one of which was over 50 years old. We used the refined 45 metagenomic elements to predict the clinical state of each independent sample (Fig. 4B). Our classifier was able to assign the clinical states of 7 samples (70%) with an accuracy of 86%, highly consistent with the classification results from the training sample set of 72 samples. Based on this, we conclude that the metagenomic elements identified in our study can be used to classify the clinical state of the skin with high accuracy.

## Discussion

The role of the human cutaneous microbiota in skin health and disease is not yet fully understood. In acne, the dominant bacterial species, *P. acnes,* has been believed to have a causal role^15^,^16^. However, the dominance of this species on healthy skin with a role in preventing the colonization of skin pathogens has long been a concern when trying to establish a link between *P. acnes* and acne pathogenesis^5^,^45^,^46^. The distinction in acne or health associations among different lineages of *P. acnes* strains was elucidated recently^5^,^19^,^20^ and partially addressed this concern. However, it is still unclear what impact on skin health or disease the skin microbiota exerts when multiple *P. acnes* strains coexist in the community.

In this study, we performed ultra-deep metagenomic shotgun sequencing of the samples collected from acne patients and young and older healthy individuals to quantitatively compare the microbiome differences between disease and health at the metagenomic level. To our knowledge, this is the first study in which the skin metagenome in a diseased state as well as in older healthy individuals has been analyzed. With a relatively large number of subjects, our ultra-deep shotgun sequencing provided a high coverage of the metagenome (equivalent to an average of 422X coverage of the *P. acnes* genome per sample), which allowed quantitative characterization of the skin microbiome. We discovered that while the skin microbiome was highly variable among individuals, there were significant differences in the relative abundances of certain species, strains, and metagenomic elements between healthy and diseased states.

We revealed a trend of higher relative abundances of *P. acnes* and *P. granulosum* in healthy individuals compared to acne patients (Fig. 1D; Table 1). This is contrary to what has been long believed and suggests a potential probiotic role of these two species in maintaining healthy skin. Early studies suggested the involvement of both *P. acnes* and *P. granulosum* in acne pathogenesis because of frequent isolations of these species from the follicles of acne patients^47^,^48^,^49^. More recently, however, using immuno-fluorescence microscopy, *P. acnes* and *P. granulosum* were identified in the follicles of healthy subjects at a similar prevalence to that of acne patients^50^. Our finding of a higher relative abundance of these species in healthy skin suggests that *P. acnes* and *P. granulosum* are essential members of the healthy skin microbial community. A beneficial role of propionibacteria in the skin microbiota is consistent with skin microbiome studies of other skin diseases, such as atopic dermatitis and psoriasis, in which the abundance of propionibacteria was reduced in the diseased states and increased after treatment^3^,^6^,^51^.

The high relative abundances of *P. acnes* and *P. granulosum* observed in healthy individuals suggest a role for these species in skin health. *Propionibacterium* species are thought to confer health benefits via the production of SCFA. SCFA possess antimicrobial properties and contribute to the acidic pH of the skin in defending against pathogens^42^,^52^,^53^. Disruption in the normal skin pH has been linked to several skin diseases, including atopic dermatitis, ichthyosis, fungal infections^54^,^55^,^56^,^57^, and acne^58^. As propionibacteria play important roles in skin health, our finding suggests that effective acne treatments should not aim to kill all *Propionibacterium* in the follicle, as practiced in most of the current acne treatments, but rather to target pathogenic strains and keep or supplement the beneficial strains to maintain a healthy state of the skin.

Compared to *P. acnes*, much less is known about the role of *P. granulosum* in skin health and disease. Limited genomic information exists for *P. granulosum*. Previous comparative analysis based on two draft genomes revealed that, compared to *P. acnes, P. granulosum* lacks virulence-related factors such as CAMP factors and sialidases, and exhibits no neuraminidase and hyaluronidase activities^26^. The bacterium may provide protection to the skin with less production of virulence-associated elements. Further study to elucidate the potential health benefits of *P. granulosum* in the skin microbiota is warranted.

In the analysis of the skin microbiome in healthy individuals, we revealed, for the first time, a positive correlation between *P. acnes* phage abundance and subject age as well as a link between phage abundance and healthy skin (Fig. 1C). This finding potentially could explain in part why the prevalence of acne declines with age^10^. The observed reduction of acne prevalence in older individuals^10^,^11^ may be a result of increased abundance of *P. acnes* phage, in addition to changes in the ecological niche such as reduced sebum and thus reduced propionibacteria load in the follicle^34^. Our findings are consistent with previous culture-based observation of a higher frequency of *P. acnes* phage isolated from healthy individuals than acne patients^29^, suggesting a role for *P. acnes* phage in acne-free skin, potentially by modulating *P. acnes* strain populations in the follicular microbiota. Despite being utilized for almost a century in Eastern Europe as a treatment for acne and other diseases^59^, few studies have been performed to understand the pray-predator interactions and dynamics between *Propionibacterium* species/strains and *P. acnes* phage populations in the skin microbial community^29^. The observation of increased phage abundance in healthy individuals merits further investigation of the role of *P. acnes* phage in skin health. Our findings suggest that it is potentially promising to design personalized and strain-specific phage-based treatment that can target specific *P. acnes* strains and thus shift the strain composition to a healthy skin microbiota^29^.

To understand the role of the skin microbiota in skin health and acne at the molecular level, we identified a robust set of metagenomic elements that are differentially abundant between the two groups and can be used as markers for classification of the clinical states of the skin. In our analysis, the clinical states of most of the individuals were accurately classified based on the 45 metagenomic elements, including the training set samples from 72 subjects and the 10 independent samples not used in the identification of the metagenomic markers. Given the large individual variations in the skin microbiome, and that acne is a multi-factorial disease with bacteria accounting for only one of the mechanisms, our finding that the metagenome of the follicular microbiota can be used to classify the host skin states is significant. Furthermore, our results suggest that it is promising to develop reliable markers based on the skin metagenome to improve diagnosis, skin health monitoring, and disease prognosis.

Among the 72 subjects in our study cohort, 20 could not be assigned with confidence using the classifier. One explanation is that acne is a disease with a wide spectrum of severity and that the skin microbiome is reflective of this large variation. Although individuals presented with acne or healthy skin at the time of sampling, the follicular microbiome might have been in the transition between the two clinical states. In support of this theory, we observed that the patients with mild acne (with acne score 1–2) were often not assigned with confidence or incorrectly assigned by our classifier. On the other hand, the majority of the patients with moderate or severe acne (with acne score 3–5) were assigned with high accuracy (Fig. 3). Consistently, in our independent validation sample set, two of the four acne patients who had a low acne score of “1” could not be assigned with confidence, while the other two patients with scores of “2” and “4”, respectively, were correctly assigned (Fig. 4B). Additionally, acne is a multi-factorial disease. Other factors including environment, host genetic predisposition, age, hormone status, and life style are also thought to play a role in acne pathogenesis^60^,^61^,^62^,^63^. Better understanding of these factors will help improve the classification of the disease.

Our findings from the metagenomic shotgun analysis of the follicular microbiome suggest that the balance between acne- and health-associated species and metagenomic elements of the skin microbiota contributes to skin health and disease. We observed that when less acne- and/or more health- associated microbial elements were present, an individual had healthy skin. On the other hand, when the balance shifted towards more acne-associated elements, the disease state prevailed. This concept of a disrupted balance in the metagenomic elements of the microbiota contributing to disease, and potentially disease severity, is largely different from the traditional definition of microorganism-associated diseases. Diseases associated with microorganisms are typically linked to the presence of a particular species or strains, ignoring other members of the resident community^64^,^65^. In this study, we raise a new viewpoint of microbial pathogenesis in acne, whereby the pathogenesis is not due to the presence of one particular species or strain but rather the balance of the overall microbial community, which leads to skin health or the development of acne. The mere presence of a disease-associated species or strain or genetic element may not necessarily confer the disease, depending on the commensal members of the community. This balance-based model for microbial pathogenesis fits well for acne, a prevalent multi-factorial disease that exhibits a wide spectrum of severity amongst the general population. We believe that this new viewpoint based on the balance of the microbiota will provide novel insights on other similar microorganism-associated diseases. Additionally, our findings suggest potential future development of personalized and targeted therapies in the treatment of acne and the maintenance of skin health by supplementing the skin microbiota with probiotic organisms to shift the balance toward a healthy microbiome.

## Materials and Methods

### Subjects

Similar to that previously described^5^, in this study, subjects with acne or healthy skin were recruited from various clinics in Southern California, USA, including private practice, managed care, and public hospital settings, as well as outside of dermatology clinics, to best represent the diversity of populations and history of medical care. The diagnosis of acne was made by board-certified dermatologists. The presence of acne was graded on a scale of 0 to 5 according to the Global Acne Severity Scale^66^. Grades were recorded for both the face and the nose separately, where zero represents healthy skin and 5 represents the most severe inflammatory cystic acne. The presence of scarring was also noted. Subjects with healthy skin were determined by board-certified dermatologists and were defined as people who had no acneiform lesions on the face, chest, and back. They were excluded if they had other skin conditions that the investigators felt would affect sampling or the microbial population on the skin. Additionally, healthy subjects were excluded if they had been previously treated for acne. Among 72 subjects recruited, 38 were acne patients with an average age of 24 years (14–37 years; Fig. S1) and had an average acne score on the face and nose of 2.4 (range 1–5) and 0.6 (range 0–2), respectively. Of the 34 healthy individuals, 30 were age-matched, with an average of 26 years old (16–38 years; Fig. S1), and four were over the age of 55, with an average of 67.5 years (55–79 years; Fig. S1). The subjects responded to a written questionnaire, administered by a physician or a well-trained study coordinator who went over each question with the subjects. Of the acne patients, 16 received acne treatments during or prior to the study, including antibiotics, benzoyl peroxide, and topical retinoids (Fig. 2). Clinical information of the study participants and the histograms of age distribution of acne patients and healthy individuals are shown in Figure S1. All subjects provided written informed consent. All protocols and consent forms were approved by both the UCLA and Los Angeles Biomedical Research Institute IRBs. The study was conducted in adherence to the Helsinki Guidelines.

### Sample collection and genomic DNA extraction

Sample collection and processing were performed as previously described^5^. Briefly, skin follicle samples were taken from the nose of the subjects using Bioré Deep Cleansing Pore Strip (Kao Brands Company, Cincinnati, OH) following the manufacturer’s instructions. Clean gloves were used for each sampling. To avoid sample cross-contamination, strict sterile working conditions were enforced, including appropriate lab-wear and routine sterilization of the working surfaces and equipment. After being removed from the nose, the strip was immediately placed into a 50 ml sterile tube and kept on ice or at 4 °C. Samples were processed within 24 hours of collection. Individual follicular plugs were manually picked from the adhesive strip with sterile forceps and placed into 2 ml sterile microcentrifuge tubes filled with ATL buffer (Qiagen) and glass beads (0.1 mm diameter) (BioSpec Products Inc., Bartlesville, OK). Cells were lysed using a beadbeater for 3 minutes at 4,800 rpm at room temperature, then centrifuged at 14,000 rpm for 5 minutes. The supernatant was retrieved and used for genomic DNA (gDNA) extraction using QIAamp DNA micro kit (Qiagen). The manufacturer protocol for extracting genomic DNA from chewing gum was used. DNA extraction reagents were routinely tested for potential contaminations using negative control extractions. Concentration of gDNA was determined using Qubit Fluorometric Quantitation (Invitrogen). Concentrations from negative control experiments were below the detectable range.

### Library construction and metagenomic shotgun sequencing

Genomic sequencing libraries were prepared using NexteraXT kit (Illumina). Briefly, 1 ng of extracted metagenomic DNA was tagmented using transposase technology. 5′ and 3′ NexteraXT dual-indices were added to uniquely barcode each library. Indexed libraries were amplified using the limited 12-cycle PCR program as instructed by the manufacturer’s guidelines. Libraries were purified with Agencourt AMPure XP magnetic beads (Beckman Coulter). Library quality and average fragment length were assessed using Bioanalyzer (Agilent Technologies). Libraries were quantified with KAPA library quantification kit (KAPA Biosystems), according to the manufacturer’s instructions. Finally, libraries were randomly pooled together and sequenced using MiSeq and/or HiSeq platforms (Illumina) with paired-end reads of 251 bp or 101 bp, respectively. Following sequencing, reads were de-multiplexed.

### Data cleaning and sequencing depth analysis

Data cleaning of the metagenomic shotgun sequences was performed following the protocol used in the HMP^21^. Briefly, low quality reads and human DNA reads were filtered out first. Human reads accounted for 43% of the sequencing data. In total, 78 Gbp of cleaned, host free sequencing data were obtained for 72 samples. The average number of mappable reads per sample was 7.2 × 10^6^ (4.0 × 10^5^–4.7 × 10^7^ reads), accounting for 72.6 ± 3.0% of the cleaned, host free data. These data were used in the downstream analyses. To ensure adequate sequencing coverage, we performed sequencing depth analyses. Rarefaction curves were plotted, demonstrating sufficient sequencing depth of *P. acnes* OGUs for functional profiling (Fig. S3).

### Taxonomic composition analysis

We determined the taxonomic composition of the microbiome by mapping metagenomic shotgun sequences against microbial reference genomes, using a method similar to that described by Schloissnig *et al*.^67^. In addition to the HMP reference genomes, we included additional *Propionibacterium* genomes and *P. acnes* phage genomes. Fungal genomes from NCBI non-redundant nucleotide database (RefSeq release 48) were also included. Bowtie2 was used in mapping the shotgun reads with 80% identity threshold^68^. The relative abundance of each taxon in each sample was calculated by counting the number of base pairs covering the genome of the organism, normalized by the genome size and the sequencing depth of the sample. To identify common taxa in the population that were either associated with acne or healthy skin, we performed statistical analysis on only the species that were identified in at least 20% of the individuals of our study cohort.

### P. acnes pan-genome and OGUs

*P. acnes* pan-genome was constructed based on the genomes of 80 *P. acnes* strains, as described by Tomida *et al*.^19^. The set of *P. acnes* OGUs was constructed similarly as described by Hansen *et al*.^69^ with minor modification. Using CD-HIT^70^, genes in *P. acnes* genomes were binned based on their nucleotide sequences with 80% identity threshold. The relative abundance of each OGU was calculated as the total base pair coverage within the OGU region normalized by the OGU length and the sequencing depth of *P. acnes* genomes in each sample. The presence of an OGU in a sample was defined as more than 1X coverage after normalization.

### Functional classification of P. acnes OGUs

*P. acnes* OGUs were annotated with KEGG orthologous groups (KOs) by mapping them to the KEGG database^71^ (version 54; e-value < 10^−5^) using BLAST, in a procedure similar to that described by Shi *et al*.^72^. The relative abundance of each functional category in each sample was calculated by summing all the functional genes involved in each functional category.

### Identification of differentially abundant OGUs and species

To minimize the potential bias due to a few individuals in the acne and healthy groupings, we used a resampling approach in identifying differentially abundant OGUs and species between acne patients and healthy individuals. We randomly selected two thirds of the samples in each group (26/38 in the acne group and 22/34 in the healthy group) and compared them to identify metagenomic elements that were significantly different in relative abundance between the two groups (*P* < 0.05, Student’s t-test). We repeated this sub-sampling and comparison 100 times and identified the OGUs and species that were differentially abundant in at least 50 of the 100 samplings.

### Classification of clinical states based on the metagenomic profile

The supervised classification was performed using the weighted gene-voting algorithm with minor modification and leave-one-out cross-validation as described by Golub *et al*.^43^ and Bleharski *et al*.^44^. The top 25 differentially abundant metagenomic elements were used for leave-one-out classification in each permutation. More details of the method are described in the Supplementary Text.

### Independent sample set for validation

An additional ten subjects were recruited and sampled, using the same sampling method described above. Four samples were collected from acne patients (average age 24.5 years), and six samples were collected from individuals with healthy skin: five age-matched subjects (average age 33.4 years) and one older subject (77 years old). Samples were sequenced to a comparable sequencing depth (average of 8.04 × 10^8^ bp per sample). The profiles of the 45 metagenomic elements identified from our study cohort were determined for each sample. Profiles based on these 45 metagenomic elements were used to classify each sample (Supplementary Text).

### Statistical analysis

Statistical analyses were performed to calculate the differences between acne patients and healthy individuals at the species and strain levels using the Wilcoxon Rank-Sum Test (U-test). We corrected for multiple testing using the Bonferroni correction. Student’s t-test with two-tailed distribution was used in statistical testing of the differences in the relative abundance of *P. acnes* OGUs between the two groups. Pearson’s correlation was used to calculate the correlation co-efficient.

### Data Availability

The sequence data from this study have been submitted to NCBI BioProject (http://www.ncbi.nlm.nih.gov/bioproject) under BioProject number 283427.

## Additional Information

**How to cite this article**: Barnard, E. *et al*. The balance of metagenomic elements shapes the skin microbiome in acne and health. *Sci. Rep.* **6**, 39491; doi: 10.1038/srep39491 (2016).

**Publisher's note:** Springer Nature remains neutral with regard to jurisdictional claims in published maps and institutional affiliations.

## Supplementary Material

Supplementary Information

## Figures and Tables

**Table 1:** Bacterial organisms identified in the follicular microbiome of the three groups.

Phylum	Reference species	Acne (n = 38)		Healthy age-matched (n = 30)		Healthy 55 + (n = 4)		Acne vs. age-matched healthy	Acne vs. all healthy
		Average relative abundance	Prevalence (percentage)	Average relative abundance	Prevalence (percentage)	Average relative abundance	Prevalence (percentage)	*P-value*	*P-value*
Actinobacteria	*P. acnes*	88.50%	38 (100%)	93.80%	30 (100%)	91.60%	4 (100%)	0.663	0.810
	*P. humerusii*	3.60%	21 (55%)	1.66%	22 (73%)	1.40%	2 (50%)	0.654	0.586
	*P. granulosum*	0.90%	9 (24%)	2.19%	17 (57%)	2.12%	3 (75%)	0.002	0.001
	*P. avidum*	0.37%	6 (16%)	0.43%	9 (30%)	0.55%	0	0.859	0.469
	Other	0.08%	—	0.07%	—	0.24%	—	—	—
Firmicutes	*S. epidermidis*	1.01%	9 (24%)	0.64%	6 (20%)	0.95%	1 (25%)	0.568	0.666
	Other	2.34%	—	0.48%	—	0.46%	—	—	—
Proteobacteria		1.88%	—	0.36%	—	1.83%	—	—	—
Cyanobacteria		1.02%	—	0.23%	—	0%	—	—	—
Bacteroidetes		0.26%	—	0.07%	—	0%	—	—	—

*Wilcoxon Rank-Sum Test (U-test) was performed on the prevalent species (found in* ≥*20% of the subject population). Significance level was adjusted to P* < *0.005 after Bonferroni correction.*

## References

[b1] Sanford J. A. & Gallo R. L. Functions of the skin microbiota in health and disease. Semin Immunol 25, 370–377, doi: 10.1016/j.smim.2013.09.005 (2013). 24268438 PMC4219649

[b2] Barnard E. & Li H. Shaping of cutaneous function by encounters with commensals. J Physiol, doi: 10.1113/JP271638 (2016). PMC5233660 26988937

[b3] Gao Z., Tseng C. H., Strober B. E., Pei Z. & Blaser M. J. Substantial alterations of the cutaneous bacterial biota in psoriatic lesions. PLoS One 3, e2719, doi: 10.1371/journal.pone.0002719 (2008). 18648509 PMC2447873

[b4] Alekseyenko A. V. . Community differentiation of the cutaneous microbiota in psoriasis. Microbiome 1, 31, doi: 10.1186/2049-2618-1-31 (2013). 24451201 PMC4177411

[b5] Fitz-Gibbon S. . Propionibacterium acnes strain populations in the human skin microbiome associated with acne. J Invest Dermatol 133, 2152–2160, doi: 10.1038/jid.2013.21 (2013). 23337890 PMC3745799

[b6] Kong H. H. . Temporal shifts in the skin microbiome associated with disease flares and treatment in children with atopic dermatitis. Genome Res 22, 850–859, doi: 10.1101/gr.131029.111 (2012). 22310478 PMC3337431

[b7] Paulino L. C., Tseng C. H., Strober B. E. & Blaser M. J. Molecular analysis of fungal microbiota in samples from healthy human skin and psoriatic lesions. J Clin Microbiol 44, 2933–2941, doi: 10.1128/JCM.00785-06 (2006). 16891514 PMC1594634

[b8] White G. M. Recent findings in the epidemiologic evidence, classification, and subtypes of acne vulgaris. J Am Acad Dermatol 39, S34–37 (1998). 9703121 10.1016/s0190-9622(98)70442-6

[b9] James W. D. Clinical practice. Acne. N Engl J Med 352, 1463–1472, doi: 10.1056/NEJMcp033487 (2005). 15814882

[b10] Collier C. N. . The prevalence of acne in adults 20 years and older. J Am Acad Dermatol 58, 56–59, doi: 10.1016/j.jaad.2007.06.045 (2008). 17945383

[b11] Perkins A. C., Maglione J., Hillebrand G. G., Miyamoto K. & Kimball A. B. Acne vulgaris in women: prevalence across the life span. J Womens Health (Larchmt) 21, 223–230, doi: 10.1089/jwh.2010.2722 (2012). 22171979

[b12] Mallon E. . The quality of life in acne: a comparison with general medical conditions using generic questionnaires. Br J Dermatol 140, 672–676 (1999). 10233319 10.1046/j.1365-2133.1999.02768.x

[b13] Dunn L. K., O’Neill J. L. & Feldman S. R. Acne in adolescents: quality of life, self-esteem, mood, and psychological disorders. Dermatol Online J 17, 1 (2011). 21272492

[b14] Zouboulis C. C. Acne and sebaceous gland function. Clin Dermatol 22, 360–366, doi: 10.1016/j.clindermatol.2004.03.004 (2004). 15556719

[b15] Bojar R. A. & Holland K. T. Acne and Propionibacterium acnes. Clin Dermatol 22, 375–379, doi: 10.1016/j.clindermatol.2004.03.005 (2004). 15556721

[b16] Leeming J. P., Holland K. T. & Cuncliffe W. J. The microbial colonization of inflamed acne vulgaris lesions. Br J Dermatol 118, 203–208 (1988). 2964856 10.1111/j.1365-2133.1988.tb01775.x

[b17] Elias P. M. The skin barrier as an innate immune element. Semin Immunopathol 29, 3–14 (2007). 17621950 10.1007/s00281-007-0060-9

[b18] Marples R. R., Downing D. T. & Kligman A. M. Control of free fatty acids in human surface lipids by Corynebacterium acnes. J Invest Dermatol 56, 127–131 (1971). 4997367 10.1111/1523-1747.ep12260695

[b19] Tomida S. . Pan-genome and comparative genome analyses of propionibacterium acnes reveal its genomic diversity in the healthy and diseased human skin microbiome. MBio 4, e00003–00013, doi: 10.1128/mBio.00003-13 (2013). 23631911 PMC3663185

[b20] Kasimatis G., Fitz-Gibbon S., Tomida S., Wong M. & Li H. Analysis of complete genomes of Propionibacterium acnes reveals a novel plasmid and increased pseudogenes in an acne associated strain. Biomed Res Int 2013, 918320, doi: 10.1155/2013/918320 (2013). 23762865 PMC3666418

[b21] Consortium H. M. P. Structure, function and diversity of the healthy human microbiome. Nature 486, 207–214, doi: 10.1038/nature11234 (2012). 22699609 PMC3564958

[b22] Mathieu A. . Life on human surfaces: skin metagenomics. PLoS One 8, e65288, doi: 10.1371/journal.pone.0065288 (2013). 23776466 PMC3680502

[b23] Oh J. . Biogeography and individuality shape function in the human skin metagenome. Nature 514, 59–64, doi: 10.1038/nature13786 (2014). 25279917 PMC4185404

[b24] Rodriguez-R L. M. & Konstantinidis K. T. Estimating coverage in metagenomic data sets and why it matters. ISME J 8, 2349–2351, doi: 10.1038/ismej.2014.76 (2014). 24824669 PMC4992084

[b25] Ordögh L. . Complete Genome Sequence of Propionibacterium avidum Strain 44067, Isolated from a Human Skin Abscess. Genome Announc 1, doi: 10.1128/genomeA.00337-13 (2013). PMC3675521 23792747

[b26] Mak T. N. . Comparative genomics reveals distinct host-interacting traits of three major human-associated propionibacteria. BMC Genomics 14, 640, doi: 10.1186/1471-2164-14-640 (2013). 24053623 PMC3848858

[b27] Butler-Wu S. M., Sengupta D. J., Kittichotirat W., Matsen F. A. & Bumgarner R. E. Genome sequence of a novel species, Propionibacterium humerusii. J Bacteriol 193, 3678, doi: 10.1128/JB.05036-11 (2011). 21571999 PMC3133328

[b28] Farrar M. D. . Genome sequence and analysis of a Propionibacterium acnes bacteriophage. J Bacteriol 189, 4161–4167, doi: 10.1128/JB.00106-07 (2007). 17400737 PMC1913406

[b29] Liu J. . The diversity and host interactions of Propionibacterium acnes bacteriophages on human skin. ISME J, doi: 10.1038/ismej.2015.47 (2015). PMC4542030 26283260

[b30] Zeeuwen P. L. . Microbiome dynamics of human epidermis following skin barrier disruption. Genome Biol 13, R101, doi: 10.1186/gb-2012-13-11-r101 (2012). 23153041 PMC3580493

[b31] Lomholt H. B. & Kilian M. Population genetic analysis of Propionibacterium acnes identifies a subpopulation and epidemic clones associated with acne. PLoS One 5, e12277, doi: 10.1371/journal.pone.0012277 (2010). 20808860 PMC2924382

[b32] McDowell A. . A novel multilocus sequence typing scheme for the opportunistic pathogen Propionibacterium acnes and characterization of type I cell surface-associated antigens. Microbiology 157, 1990–2003, doi: 10.1099/mic.0.049676-0 (2011). 21511767

[b33] McGinley K. J. & Webster G. F. & Leyden, J. J. Regional variations of cutaneous propionibacteria. Appl Environ Microbiol 35, 62–66 (1978). 623473 10.1128/aem.35.1.62-66.1978 PMC242779

[b34] Yamamoto A., Serizawa S., Ito M. & Sato Y. Effect of aging on sebaceous gland activity and on the fatty acid composition of wax esters. J Invest Dermatol 89, 507–512 (1987). 3668294 10.1111/1523-1747.ep12461009

[b35] Gao Z., Tseng C. H., Pei Z. & Blaser M. J. Molecular analysis of human forearm superficial skin bacterial biota. Proc Natl Acad Sci USA 104, 2927–2932, doi: 10.1073/pnas.0607077104 (2007). 17293459 PMC1815283

[b36] Costello E. K. . Bacterial community variation in human body habitats across space and time. Science 326, 1694–1697, doi: 10.1126/science.1177486 (2009). 19892944 PMC3602444

[b37] Chatterjee C., Paul M., Xie L. & van der Donk W. A. Biosynthesis and mode of action of lantibiotics. Chem Rev 105, 633–684, doi: 10.1021/cr030105v (2005). 15700960

[b38] Attaiech L. . Role of the single-stranded DNA-binding protein SsbB in pneumococcal transformation: maintenance of a reservoir for genetic plasticity. PLoS Genet 7, e1002156, doi: 10.1371/journal.pgen.1002156 (2011). 21738490 PMC3128108

[b39] Lee S. W. . Discovery of a widely distributed toxin biosynthetic gene cluster. Proc Natl Acad Sci USA 105, 5879–5884, doi: 10.1073/pnas.0801338105 (2008). 18375757 PMC2311365

[b40] Nizet V. . Genetic locus for streptolysin S production by group A streptococcus. Infect Immun 68, 4245–4254 (2000). 10858242 10.1128/iai.68.7.4245-4254.2000 PMC101736

[b41] Kjos M., Snipen L., Salehian Z., Nes I. F. & Diep D. B. The abi proteins and their involvement in bacteriocin self-immunity. J Bacteriol 192, 2068–2076, doi: 10.1128/JB.01553-09 (2010). 20154137 PMC2849437

[b42] Shu M. . Fermentation of Propionibacterium acnes, a commensal bacterium in the human skin microbiome, as skin probiotics against methicillin-resistant Staphylococcus aureus. PLoS One 8, e55380, doi: 10.1371/journal.pone.0055380 (2013). 23405142 PMC3566139

[b43] Golub T. R. . Molecular classification of cancer: class discovery and class prediction by gene expression monitoring. Science 286, 531–537 (1999). 10521349 10.1126/science.286.5439.531

[b44] Bleharski J. R. . Use of genetic profiling in leprosy to discriminate clinical forms of the disease. Science 301, 1527–1530, doi: 10.1126/science.1087785 (2003). 12970564

[b45] Leeming J. P., Holland K. T. & Cunliffe W. J. The microbial ecology of pilosebaceous units isolated from human skin. J Gen Microbiol 130, 803–807 (1984). 6234376 10.1099/00221287-130-4-803

[b46] Jahns A. C. . An increased incidence of Propionibacterium acnes biofilms in acne vulgaris: a case-control study. Br J Dermatol 167, 50–58, doi: 10.1111/j.1365-2133.2012.10897.x (2012). 22356121

[b47] Fleming A. On the etiology of acne vulgaris and its treatment by vaccines. 1, 1035–1038 (1909).

[b48] Marples R. R., McGinley K. J. & Mills O. H. Microbiology of comedones in acne vulgaris. J Invest Dermatol 60, 80–83 (1973). 4266324 10.1111/1523-1747.ep12724149

[b49] Gehse M., Höffler U., Gloor M. & Pulverer G. Propionibacteria in patients with acne vulgaris and in healthy persons. Arch Dermatol Res 275, 100–104 (1983). 6223602 10.1007/BF00412883

[b50] Jahns A. C., Eilers H., Ganceviciene R. & Alexeyev O. A. Propionibacterium species and follicular keratinocyte activation in acneic and normal skin. Br J Dermatol 172, 981–987, doi: 10.1111/bjd.13436 (2015). 25279837

[b51] Shi B. . The skin microbiome is different in pediatric versus adult atopic dermatitis. J Allergy Clin Immunol doi: 10.1016/j.jaci.2016.04.053 (2016). 10.1016/j.jaci.2016.04.053 PMC5235385 27474122

[b52] Rothman S. & Lorincz A. L. Defense mechanisms of the skin. Annu Rev Med 14, 215–242, doi: 10.1146/annurev.me.14.020163.001243 (1963). 13975379

[b53] Ushijima T., Takahashi M. & Ozaki Y. Acetic, propionic, and oleic acid as the possible factors influencing the predominant residence of some species of Propionibacterium and coagulase-negative Staphylococcus on normal human skin. Can J Microbiol 30, 647–652 (1984). 6744125 10.1139/m84-096

[b54] Schmid-Wendtner M. H. & Korting H. C. The pH of the skin surface and its impact on the barrier function. Skin Pharmacol Physiol 19, 296–302, doi: 10.1159/000094670 (2006). 16864974

[b55] Schnyder U. W., Gloor M. & Taugner M. [Socio-medical significance of alkalie resistance, alkalie neutralization and skin-surface lipid content in atopic neurodermatitis and ichthyosis vulgaris (author’s transl)]. Berufsdermatosen 25, 101–107 (1977). 907654

[b56] Epprecht R. [Electrometric determination of the skin surface pH in healthy and eczema patients with special reference to acid neutralization capacity]. Dermatologica 111, 204–223 (1955). 13277369

[b57] Runeman B., Faergemann J. & Larkö O. Experimental Candida albicans lesions in healthy humans: dependence on skin pH. Acta Derm Venereol 80, 421–424 (2000). 11243634 10.1080/000155500300012819

[b58] Korting H. C. . The influence of the regular use of a soap or an acidic syndet bar on pre-acne. Infection 23, 89–93 (1995). 7622270 10.1007/BF01833872

[b59] Sulakvelidze A., Alavidze Z. & Morris J. G. Bacteriophage therapy. Antimicrob Agents Chemother 45, 649–659, doi: 10.1128/AAC.45.3.649-659.2001 (2001). 11181338 PMC90351

[b60] Kang D., Shi B., Erfe M. C., Craft N. & Li H. Vitamin B12 modulates the transcriptome of the skin microbiota in acne pathogenesis. Sci Transl Med 7, 293ra103, doi: 10.1126/scitranslmed.aab2009 (2015). PMC6049814 26109103

[b61] Bataille V., Snieder H., MacGregor A. J., Sasieni P. & Spector T. D. The influence of genetics and environmental factors in the pathogenesis of acne: a twin study of acne in women. J Invest Dermatol 119, 1317–1322, doi: 10.1046/j.1523-1747.2002.19621.x (2002). 12485434

[b62] Wolf R., Matz H. & Orion E. Acne and diet. Clin Dermatol 22, 387–393, doi: 10.1016/j.clindermatol.2004.03.007 (2004). 15556724

[b63] Zouboulis C. C. [Acne vulgaris. The role of hormones]. Hautarzt 61, 107–108, 110–104, doi: 10.1007/s00105-009-1830-1 (2010). 20107754

[b64] Tarr P. I., Gordon C. A. & Chandler W. L. Shiga-toxin-producing Escherichia coli and haemolytic uraemic syndrome. Lancet 365, 1073–1086, doi: 10.1016/S0140-6736(05)71144-2 (2005). 15781103

[b65] Chambers H. F. & Deleo F. R. Waves of resistance: Staphylococcus aureus in the antibiotic era. Nat Rev Microbiol 7, 629–641, doi: 10.1038/nrmicro2200 (2009). 19680247 PMC2871281

[b66] Dréno B. . Development and evaluation of a Global Acne Severity Scale (GEA Scale) suitable for France and Europe. J Eur Acad Dermatol Venereol 25, 43–48, doi: 10.1111/j.1468-3083.2010.03685.x (2011). 20456560

[b67] Schloissnig S. . Genomic variation landscape of the human gut microbiome. Nature 493, 45–50, doi: nature11711 [pii]10.1038/nature11711 (2013). 23222524 10.1038/nature11711 PMC3536929

[b68] Langmead B. & Salzberg S. L. Fast gapped-read alignment with Bowtie 2. Nat Methods 9, 357–359, doi: 10.1038/nmeth.1923 (2012). 22388286 PMC3322381

[b69] Hansen E. E. . Pan-genome of the dominant human gut-associated archaeon, Methanobrevibacter smithii, studied in twins. Proc Natl Acad Sci USA 108 Suppl 1, 4599–4606, doi: 10.1073/pnas.1000071108 (2011). 21317366 PMC3063581

[b70] Li W., Jaroszewski L. & Godzik A. Clustering of highly homologous sequences to reduce the size of large protein databases. Bioinformatics 17, 282–283 (2001). 11294794 10.1093/bioinformatics/17.3.282

[b71] Kanehisa M., Goto S., Kawashima S., Okuno Y. & Hattori M. The KEGG resource for deciphering the genome. Nucleic Acids Res 32, D277–280, doi: 10.1093/nar/gkh06332/suppl_1/D277[pii] (2004). 14681412 PMC308797

[b72] Shi B. . Dynamic changes in the subgingival microbiome and their potential for diagnosis and prognosis of periodontitis. MBio 6, e01926–01914, doi: 10.1128/mBio.01926-14 (2015). 25691586 PMC4337560

