# Author Correction: The balance of metagenomic elements shapes the skin microbiome in acne and health

**DOI:** 10.1038/s41598-020-62764-8

**Published:** 2020-04-02

**Authors:** Emma Barnard, Baochen Shi, Dezhi Kang, Noah Craft, Huiying Li

**Affiliations:** 1 Department of Molecular and Medical Pharmacology, Crump Institute for Molecular Imaging, David Geffen School of Medicine, UCLA, Los Angeles, California USA; 2 Los Angeles Biomedical Research Institute at Harbor-UCLA Medical Center, Torrance, California USA; 3 UCLA-DOE Institute for Genomics and Proteomics, Los Angeles, California USA

Correction to: *Scientific Reports* 10.1038/srep39491, published online 21 December 2016

The Article contains an error in the Data Availability section, where the accession code is missing the BioProject prefix “PRJNA”. The correct accession is PRJNA283427. As such, this section should read:

Data Availability

The sequence data from this study have been submitted to NCBI BioProject (http://www.ncbi.nlm.nih.gov/bioproject) under BioProject number PRJNA283427.

