# Supplementary material for: The balance of metagenomic elements shapes the skin microbiome in acne and health
Source: Sci Rep. 2016 Dec 21;6:39491. doi: 10.1038/srep39491 (PMC5175143; doi:10.1038/srep39491)
Supplement: Supplementary Information [file srep39491-s1.pdf]

Supplementary Information

Title:

The balance of metagenomic elements shapes the skin microbiome in acne and health

Authors:

Emma Barnard, Baochen Shi, Dezhi Kang, Noah Craft, and Huiying Li

Supplementary Text

Observed low abundance of fungal species in the skin follicle

We identified the sequencing reads mapped to six fungal species (Fig. 1B). Compared to bacteria, the reads mapped to fungal species in our metagenomic shotgun data were markedly fewer (Fig. 1A). When we combined all the reads mapped to fungal species, the maximum genome coverage for any one fungal species was 0.56X (*Malassezia globosa*) (Fig. 1B). One explanation for the observed low abundance of fungi in our dataset is that the DNA extraction method used was not optimized for retrieval of fungal DNA. Extraction of fungal DNA requires harsh methods due to the complex nature of the fungal cell wall. Another explanation is that the fungal burden of the skin follicle may be naturally low. A recent skin metagenomic study, optimized for fungal DNA recovery, revealed low fungal biomass across sebaceous sites [1]. Additionally, a previous study, which visualized bacteria inside the sebaceous follicle, was unable to detect any fungal species in either acne or healthy skin follicles from the face [2]. Fungi have previously been thought to play a role in acne, having been isolated from the follicles of the upper back skin of acne patients [3]. With deep sequencing of the follicular microbiota, we identified fungi in the skin follicle with low prevalence and abundance, and found no significant difference between acne patients and healthy individuals.

The role of minor bacterial taxa in the skin follicle

Previous studies have highlighted the role of lowly abundant species in disrupting the existing host-microbial homeostasis in other inflammatory conditions [4]. Deep sequencing allowed us to identify and compare the prevalence and abundance of minor taxa between acne patients and healthy individuals. In the skin follicle, we found low relative abundances of Firmicutes, Proteobacteria, Cyanobacteria, and Bacteroidetes (Fig. 1D). Among them, Firmicutes and Proteobacteria species were more abundant in acne patients (Fig. 1D; Table 1). One explanation for the observation is that lesional skin, as a result of inflammation, may provide an ideal niche for the growth of non-skin commensal species. Changes in the microbiota at damaged skin sites have been previously reported, with Gram-negative and Enterococci species shown to be twice as likely to colonize at damaged skin sites [5]. Cyanobacteria are not considered normal

inhabitants of the skin in general, yet have been detected at low abundances at sebaceous sites of healthy individuals and shown persistent on palm skin [6-8]. The variable presence, low prevalence, and low abundance of the minor taxa in both acne patients and, albeit lower, in healthy individuals suggest that these species are less likely to be causative. Furthermore, the reduced relative abundance of *Propionibacteria* in acne patients compared to healthy individuals may lead to an increase in the skin pH, disrupting the healthy skin environment, subsequently facilitating colonization of pathogens and other opportunistic organisms.

Classification of clinical states using weighted gene voting algorithm

We performed a supervised class prediction analysis. Differentially abundant metagenomic elements, including *P. acnes* OGUs and bacterial species, were determined between two groups of samples (healthy and acne). We employed a method similar to that described by Golub *et al.* and Bleharski *et al.* [9,10], using the formula:

$$PS = \frac{\sum_{g=1}^{n} t_g \times \left(X_g - \left(\frac{\mu_{1g} + \mu_{2g}}{2} \right) \right)}{\sum_{g=1}^{n} \left| t_g \times \left(X_g - \left(\frac{\mu_{1g} + \mu_{2g}}{2} \right) \right) \right|}$$

where PS is the prediction strength, a measure of the relative margin of victory of the vote, X_g is the relative abundance of the metagenomic element (g) in the tested sample, μ_{1g} and μ_{2g} are the means of the relative abundances of metagenomic element (g) in the two groups, and t_g is the t-test score of the metagenomic element (g) when its relative abundance is compared between the two groups using the Student's t-test. The numerator indicates the difference between the winning and losing classes, and the denominator indicates the totals for the winning and losing classes.

Supplementary References

1 Oh, J. *et al.* Biogeography and individuality shape function in the human skin metagenome. *Nature* **514**, 59-64, doi:10.1038/nature13786 (2014).

2 Jahns, A. C. *et al.* An increased incidence of Propionibacterium acnes biofilms in acne vulgaris: a case-control study. *Br J Dermatol* **167**, 50-58, doi:10.1111/j.1365-2133.2012.10897.x (2012).

3 Leeming, J. P., Holland, K. T. & Cunliffe, W. J. The microbial ecology of pilosebaceous units isolated from human skin. *J Gen Microbiol* **130**, 803-807 (1984).

4 Hajishengallis, G. *et al.* Low-abundance biofilm species orchestrates inflammatory periodontal disease through the commensal microbiota and complement. *Cell Host Microbe* **10**, 497-506, doi:10.1016/j.chom.2011.10.006 (2011).

5 Larson, E. L. *et al.* Changes in bacterial flora associated with skin damage on hands of health care personnel. *Am J Infect Control* **26**, 513-521 (1998).

6 Grice, E. A. *et al.* Topographical and temporal diversity of the human skin microbiome. *Science* **324**, 1190-1192, doi:10.1126/science.1171700 (2009).

7 Grice, E. A. & Segre, J. A. The skin microbiome. *Nat Rev Microbiol* **9**, 244-253, doi:10.1038/nrmicro2537 (2011).

8 Caporaso, J. G. *et al.* Moving pictures of the human microbiome. *Genome Biol* **12**, R50, doi:10.1186/gb-2011-12-5-r50 (2011).

9 Golub, T. R. *et al.* Molecular classification of cancer: class discovery and class prediction by gene expression monitoring. *Science* **286**, 531-537 (1999).

10 Bleharski, J. R. *et al.* Use of genetic profiling in leprosy to discriminate clinical forms of the disease. *Science* **301**, 1527-1530, doi:10.1126/science.1087785 (2003).

11 Marples, R. R., McGinley, K. J. & Mills, O. H. Microbiology of comedones in acne vulgaris. *J Invest Dermatol* **60**, 80-83 (1973).

12 Knutson, D. D. Ultrastructural observations in acne vulgaris: the normal sebaceous follicle and acne lesions. *J Invest Dermatol* **62**, 288-307 (1974).

13 Cunliffe, W. J. & Gould, D. J. Prevalence of facial acne vulgaris in late adolescence and in adults. *Br Med J* **1**, 1109-1110 (1979).

14 Knop, J., Ollefs, K. & Frosch, P. J. Anti-P. acnes antibody in comedonal extracts. *J Invest Dermatol* **80**, 9-12 (1983).

15 Sheehan-Dare, R. A., Hughes, B. R. & Cunliffe, W. J. Clinical markers of androgenicity in acne vulgaris. *Br J Dermatol* **119**, 723-730 (1988).

16 Vahlquist, A., Rollman, O., Holland, D. B. & Cunliffe, W. J. Isotretinoin treatment of severe acne affects the endogenous concentration of vitamin A in sebaceous glands. *J Invest Dermatol* **94**, 496-498 (1990).

17 Eady, E. A. et al. The effects of acne treatment with a combination of benzoyl peroxide and erythromycin on skin carriage of erythromycin-resistant propionibacteria. *Br J Dermatol* **134**, 107-113 (1996).

18 Pappas, A., Johnsen, S., Liu, J. C. & Eisinger, M. Sebum analysis of individuals with and without acne. *Dermatoendocrinol* **1**, 157-161 (2009).

A

Clinical group	Acne	Healthy (age-matched)	Healthy 55+
Number of subjects	38	30	4
**Gender n (%)**			
Males	13 (34%)	16 (53%)	2(50%)
Females	25 (66%)	14 (47%)	2(50%)
**Age (years)**			
Range	14-37	16-38	55-79
Average	24	26	67.5
Median	24.5	27	68
**Ethnicity/Race n (%)**			
American Indian	1 (2.6%)	0	0
Asian	12 (31.6%)	10 (33%)	2 (50%)
Black	6 (15.8%)	0	0
Hispanic	5 (13%)	10 (33%)	0
White	11 (29%)	8 (27%)	2 (50%)
More than one race	3 (8%)	2 (7%)	0

B

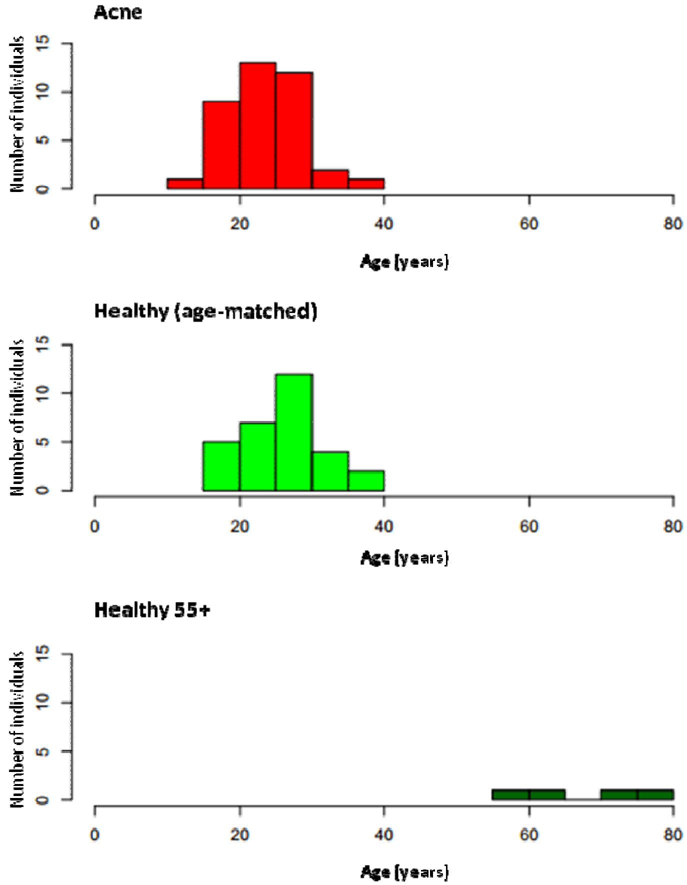

Supplementary Fig. S1: Clinical information of the study participants. **A**) Clinical information of the acne patients (n=38), age-matched healthy individuals (n=30), and healthy individual with age over 55 (n=4). **B**) Histograms of age distribution within each subject group. Similar to previous studies[11-18], we include both teenagers and young adults in acne and healthy age-matched groups.

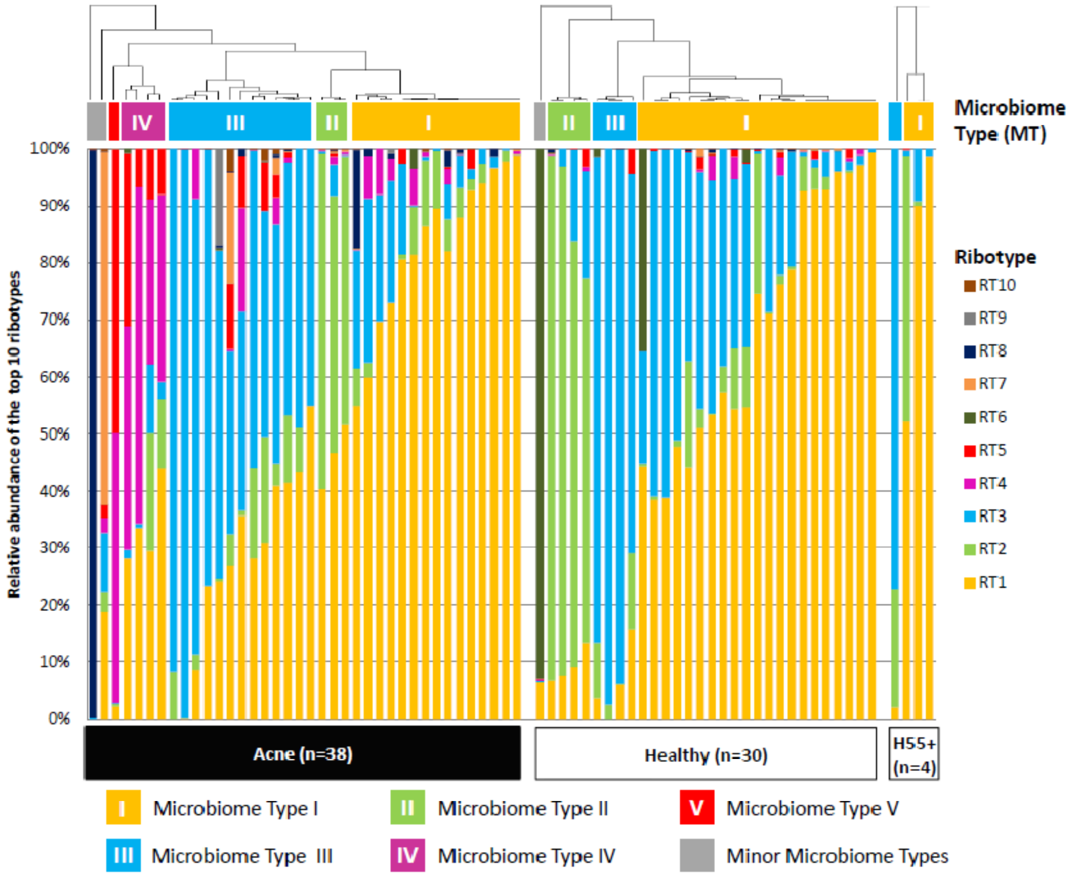

Supplementary Fig. S2: *P. acnes* strain populations are different between acne patients and healthy individuals. Each column represents the relative abundances of the top ten *P. acnes* ribotypes in each individual. Acne patients, age-matched healthy individuals, and healthy individuals with age over 55 were included. Individuals often harbor more than one ribotype (an average of 2.3 ribotypes per person). Individuals were clustered based on the composition of the top ten ribotypes. Microbiome Types IV and V were found in the acne patients, but not in the healthy individuals.

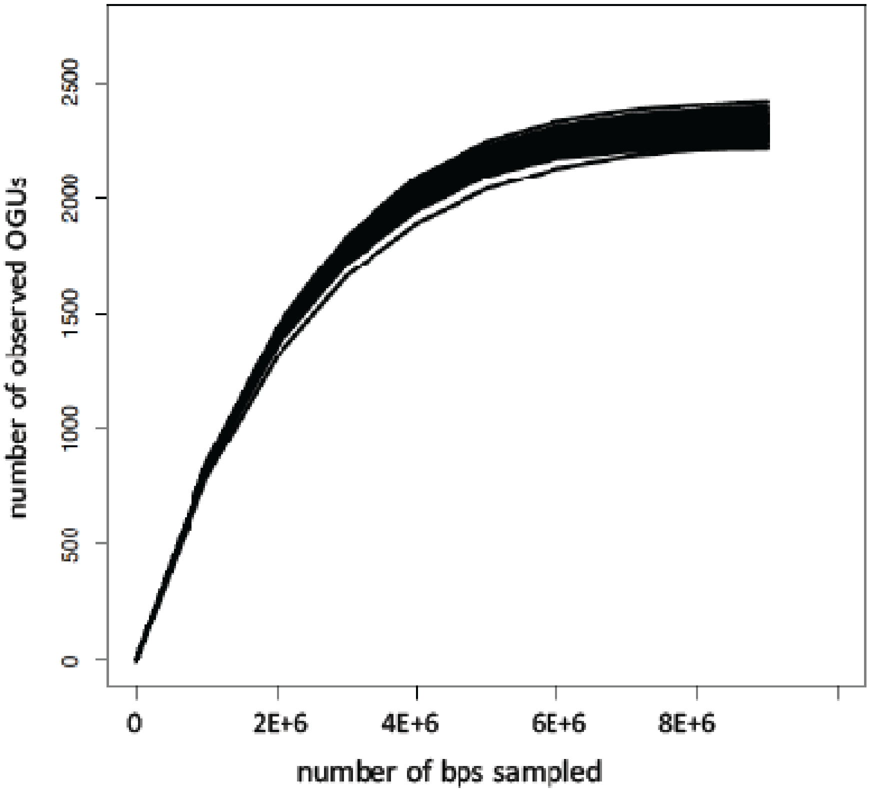

Supplementary Fig. S3: Rarefaction curves indicate sufficient sequencing depth of all samples. The rarefaction curves of the 72 samples all reached the plateau, suggesting that the sequencing depth of all the samples was sufficient for detecting *P. acnes* OGUs and for comparative functional profiling. The sequencing depth ranged from $6.9 \times 10^7 - 4.8 \times 10^9$ base pairs per sample. The rarefaction curves beyond 9×10^6 base pairs are not shown.

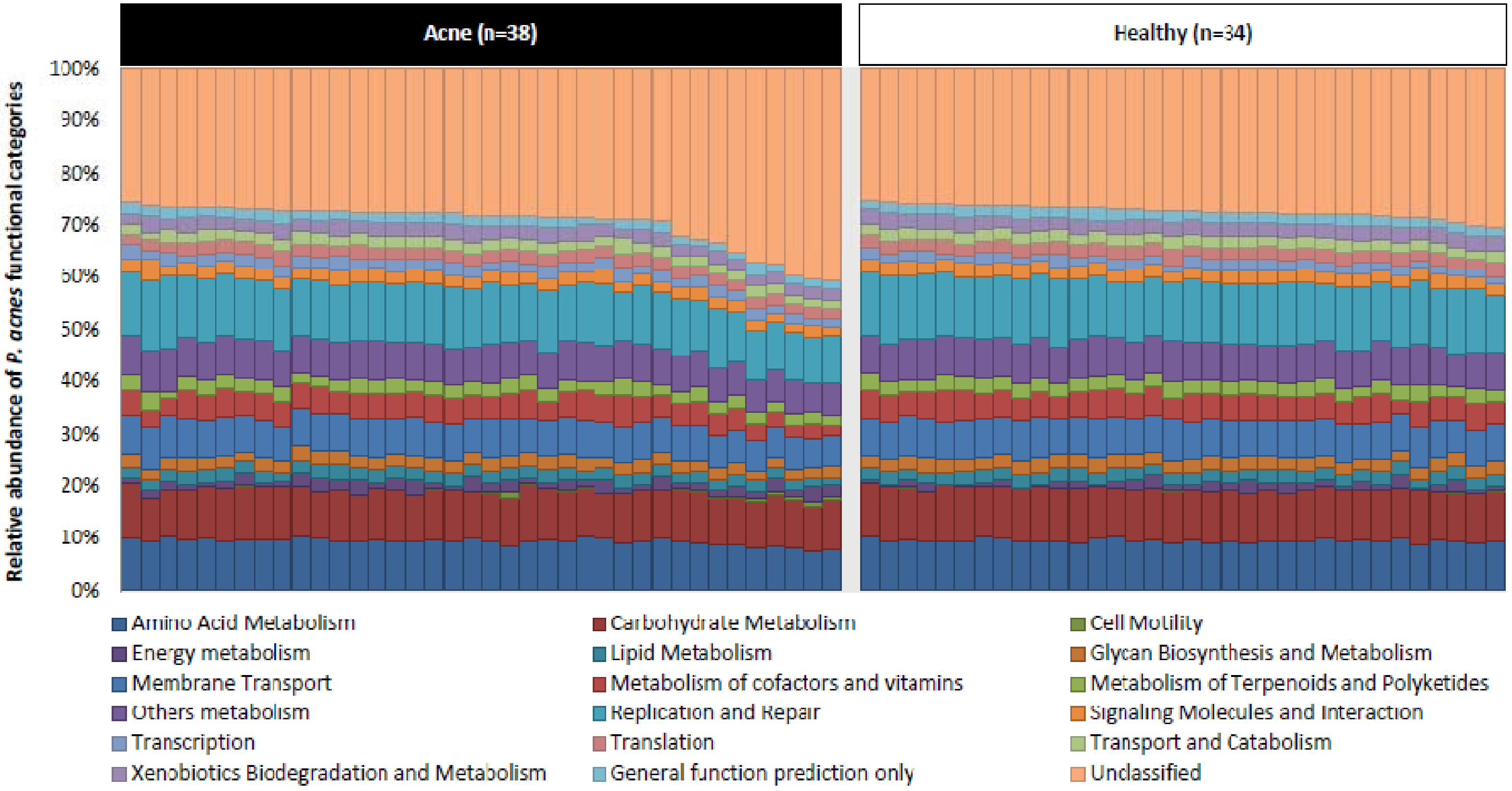

Supplementary Fig. S4: Functional profiles of the differentially abundant OGUs in acne patients and healthy individuals. *P. acnes* OGUs that were differentially abundant between acne patients and healthy individuals were assigned to functional categories. The functional profiles from the acne patients varied between individuals with higher abundances of unclassified genes, while in healthy individuals the functional profiles remained relatively stable across individuals.

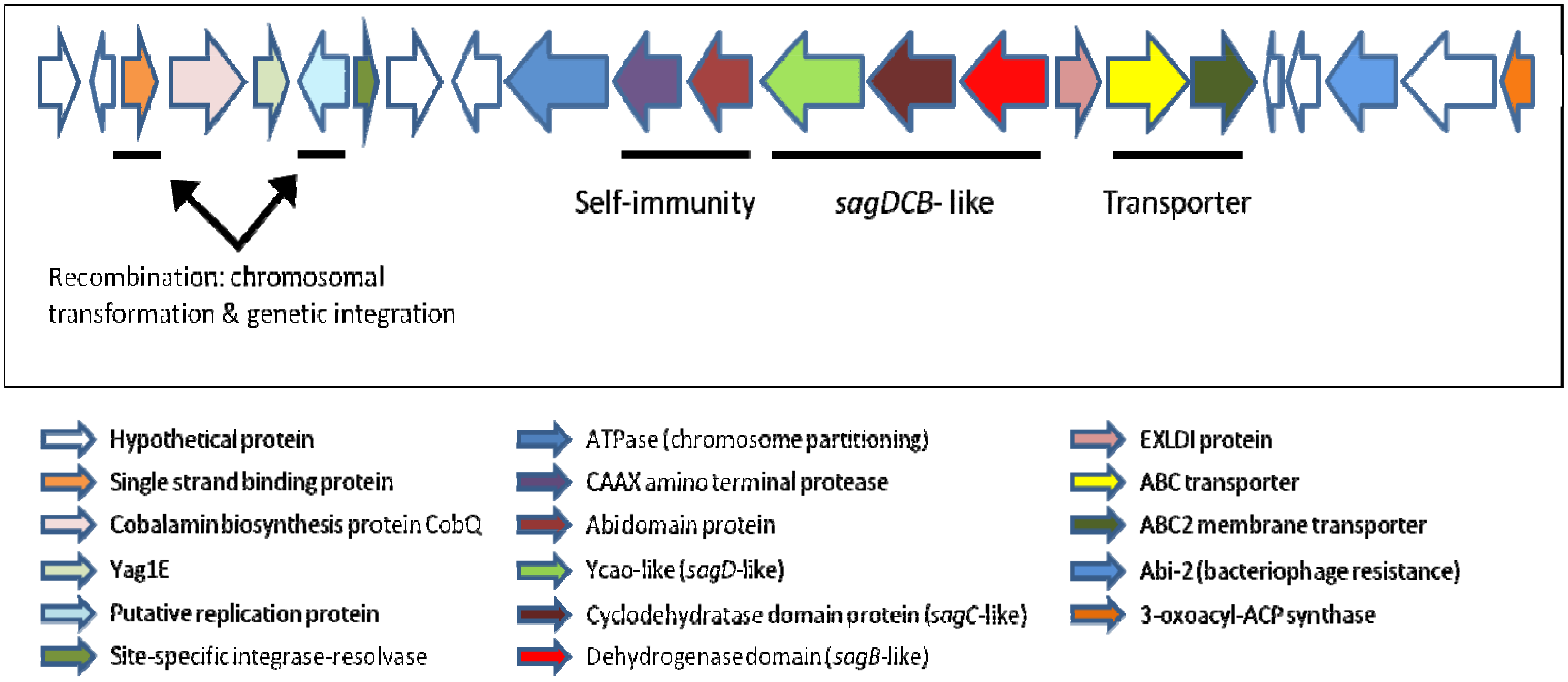

Supplementary Fig. S5: Locus 2 encodes virulence-related genes. Locus 2 is a 20 Kb genomic island predominantly found in *P. acnes* clade IA-2 strains, RT4 and RT5. Locus 2 encodes 23 ORFs including a cluster of Streptolysin S-associated genes (*sag*) involved in biosynthesis and transport of bacterial toxins as well as self-immunity. Relative gene length and directionality for each gene encoded in locus 2 of *P. acnes* HL096PA1 is shown.

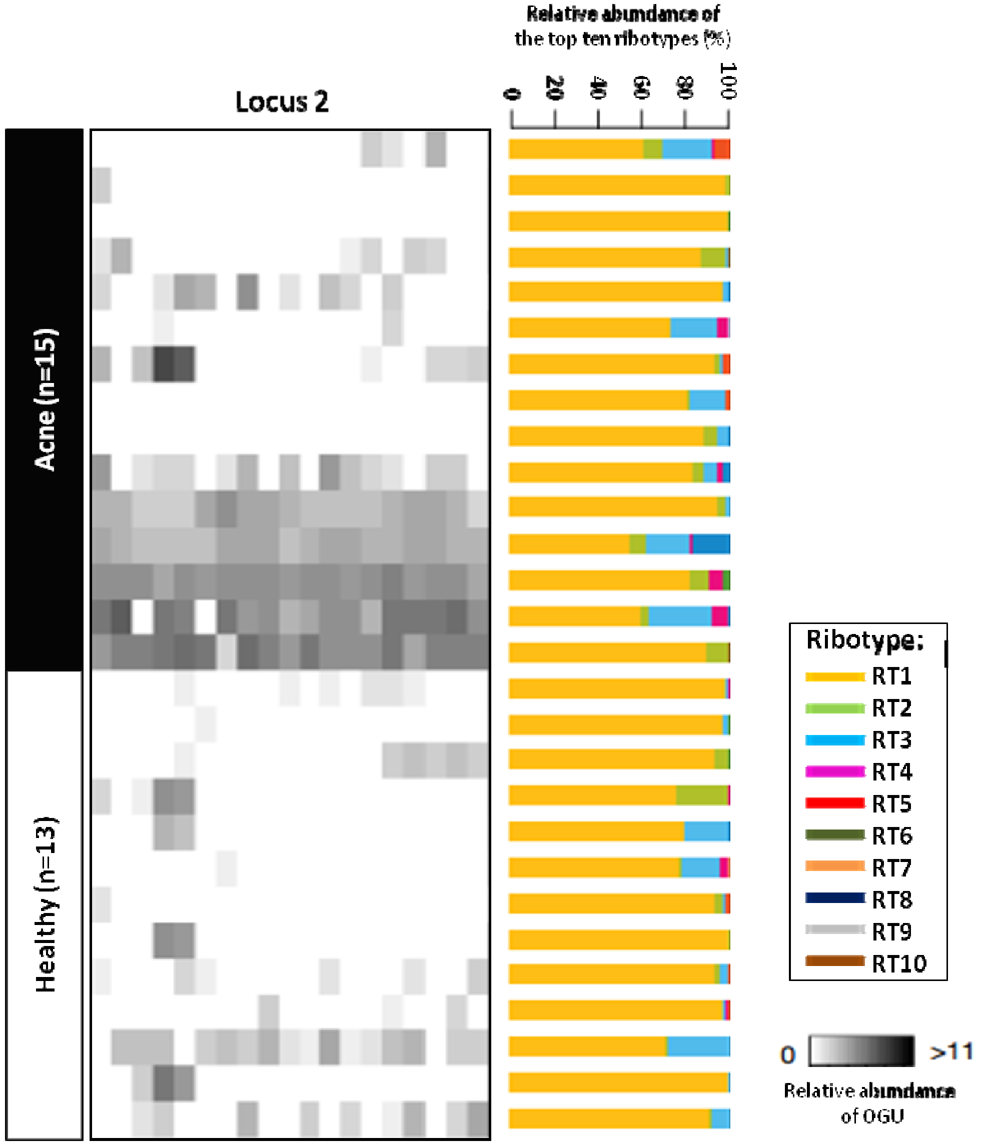

Supplementary Fig. S6: Locus 2 was more abundant in the acne patients with MTI than in the healthy individuals with MT1. The relative abundance of locus 2 in 15 acne patients and 13 healthy individuals with MTI is shown. Each column represents one of the 19 OGUs of locus 2, which were significantly different between acne patients and healthy individuals, plotted in the order of their genomic positions in the locus (as listed in Supplementary Table S1). A significant increase (*P*=0.02) of the relative abundances of locus 2 OGUs was observed in acne patients compared to healthy individuals with MTI. The top ten ribotype composition for each individual is also shown.

Supplementary Table S1: Differentially abundant *P. acnes* OGUs between acne patients and healthy individuals.

P. acnes Gene ID	Predicted function	Clinical Association
PAGK_0029	Transcriptional regulator	Acne
PAGK_0033	Acetyltransferase	Health
PAGK_0136	Putative glycosyl transferase	Health
PAGK_0138	Chain-length determining protein	Health
PAGK_0160 (Locus 2)	Hypothetical protein	Acne
PAGK_0161 (Locus 2)	Hypothetical protein	Acne
PAGK_0162 (Locus 2)	Single-strand binding protein (Ssb)	Acne
PAGK_0163 (Locus 2)	Cobalamin biosynthesis protein CobQ/partitioning protein RepA	Acne
PAGK_0164 (Locus 2)	Yag1E	Acne
PAGK_0165 (Locus 2)	Putative replication protein	Acne
PAGK_0166 (Locus 2)	Site-specific integrase-resolvase	Acne
PAGK_0167 (Locus 2)	Hypothetical protein	Acne
PAGK_0168 (Locus 2)	Hypothetical protein	Acne
PAGK_0169 (Locus 2)	ATPase/chromosome partitioning	Acne
PAGK_0170 (Locus 2)	Caax amino terminal protease	Acne
PAGK_0171 (Locus 2)	Abi domain protein	Acne
PAGK_0172 (Locus 2)	YcaO-like family protein (SagD-like)	Acne
PAGK_0173 (Locus 2)	Cyclodehydratase protein (SagC-like)	Acne
PAGK_0174 (Locus 2)	Oxidoreductase protein (SagB-like)	Acne
PAGK_0175 (Locus 2)	EXLDI domain protein	Acne
PAGK_0176 (Locus 2)	ABC transporter/ATP-binding protein	Acne
PAGK_0177 (Locus 2)	ABC-2 type membrane transporter	Acne
PAGK_0178 (Locus 2)	Hypothetical protein	Acne
PAGK_0198	ABC transporter	Acne
PAGK_0374	2-C-methyl-D-erythritol 4-phosphate	Health
PAGK_0387	L-asparaginase I	Acne
PAGK_0505	Sugar transporter family protein	Health
PAGK_0665	Glutamate dehydrogenase	Health
PAGK_0667	Putative 6-aminohexanoate-dimer hydrolase	Health
PAGK_0687	Hypothetical protein	Health
PAGK_0712	Hypothetical protein	Health
PAGK_0754	DNA processing / uptake protein	Health
PAGK_0755	Methylated-DNA--protein-cysteine	Health
PAGK_0768	Translation initiation factor IF-3	Health
PAGK_0793	Site-specific tyrosine recombinase XerD	Health
PAGK_0820	Putative acyltransferase	Health
PAGK_0821	D-alanine--D-alanine ligase	Health
PAGK_0880	Putative ATP-binding ABC transporter protein	Health
PAGK_0896	Diaminopimelate decarboxylase	Health
PAGK_0979	Hypothetical protein	Health
PAGK_0983	Hypothetical protein	Health
PAGK_0985	Metallo-beta-lactamase superfamily protein	Health
PAGK_1034	Hypothetical protein	Health
PAGK_1035	Cobalamin independent methionine synthase	Health

PAGK_1039	Putative alpha-amylase	Health
PAGK_1043	Putative pyrophosphohydrolase	Health
PAGK_1113	Putative aminooxidase	Health
PAGK_1168	2-dehydropantoate 2-reductase	Health
PAGK_1302	GTPase ObgE	Health
PAGK_1393	Putative helicase protein	Health
PAGK_1606	Ferrous iron transport protein B	Health
PAGK_1635	Hypothetical protein	Health
PAGK_1813	Exodeoxyribonuclease III	Health
PAGK_1815	Hypothetical protein	Health
PAGK_1820	2-oxoglutarate ferredoxin oxidoreductase	Health
PAGK_2086	Potassium transporter	Health
PAGK_2104	Thiazolylpeptide-type bacteriocin precursor	Acne
PAGK_2105	ABC transporter/ATP-binding protein	Acne
PAGK_2133	Single-strand DNA-binding protein	Health
PAGK_2214	Glycerol uptake facilitator protein	Health
PAGK_2332 (Locus 3)	Plasmid partitioning protein ParA	Acne
HMPREF9572DFT_Contig29.2.Glimmer3.p5_hybrid.274	Hypothetical protein	Health
